# Association of inflammatory cytokines expression in cerebrospinal fluid with the severity and prognosis of spontaneous intracerebral hemorrhage

**DOI:** 10.1186/s12883-023-03487-x

**Published:** 2024-01-02

**Authors:** Tianyan Gu, Jingyu Pan, Ling Chen, Kai Li, Li Wang, Zhihao Zou, Qinghai Shi

**Affiliations:** 1 Graduate School of Xinjiang Medical University, Urumqi, Xinjiang 830000 China; 2 Medical College of Shihezi University, Shihezi, Xinjiang 832000 China; 3 Clinical Laboratory Diagnostic Center, General Hospital of Xinjiang Military Command, Urumqi, Xinjiang 830000 China; 4 Department of Neurosurgery, General Hospital of Xinjiang Military Command, Urumqi, Xinjiang 830000 China

**Keywords:** Intracerebral Hemorrhage, Inflammation, Cerebrospinal fluid, Cytokines

## Abstract

**Objective:**

To investigate the potential diagnostic and prognostic implications of inflammatory cytokine levels in the cerebrospinal fluid (CSF) of patients with spontaneous intracerebral hemorrhage (SICH) upon their initial hospital admission.

**Methods:**

Our cohort included 100 patients diagnosed with acute SICH, presenting to the Department of Neurosurgery. Additionally, we recruited 50 individuals without central nervous system (CNS) pathology, treated concurrently at our facility, as controls. CSF samples, collected upon hospital entry, were quantitatively assessed for 10 inflammatory cytokines using the Mesoscale Discovery Platform (MSD, Rockville, MD, USA) electrochemiluminescence technology, followed by validation through enzyme-linked immunosorbent assay (ELISA).

**Results:**

We observed a marked elevation of IL-6, IL-8, IL-10, and TNF-α in the CSF of the SICH subgroup compared to controls. Higher Glasgow Coma Scale (GCS) scores in SICH patients corresponded with lower CSF concentrations of IL-6, IL-8, IL-10, and TNF-α, indicating an inverse relationship. Notably, CSF inflammatory cytokine levels were consistently higher in SICH patients with hydrocephalus than in those without. Increases in IL-6, IL-8, IL-10, and TNF-α in the CSF were notably more pronounced in the poor prognosis group (Glasgow Outcome Scale, GOS 1–3) compared to those with a favorable prognosis (GOS 4–5). The AUC values for these cytokines in predicting SICH prognosis were 0.750, 0.728, 0.717, and 0.743, respectively.

**Conclusions:**

Initial CSF levels of IL-6, IL-8, IL-10, and TNF-α upon admission provide significant insights into the severity of neural damage and are robust indicators for prognosis in SICH patients.

## Introduction

Intracerebral hemorrhage (ICH) is a critically severe and often fatal type of stroke, comprising approximately 10–15% of all strokes, thus representing a significant clinical emergency [1]. Despite advancements in clinical management, especially in hematoma surgical interventions, the prognosis for ICH patients is alarmingly poor, with mortality rates nearing 50%. This high mortality is primarily due to the combined effects of primary and secondary brain injuries [2]. Primary brain injuries typically arise from hematoma compression and acute hydrocephalus caused by cerebrovascular rupture and subsequent hemorrhage. Concurrently, secondary brain injuries result from the crucial role of the inflammatory response, driven by microglia activation [3]. The breakdown of erythrocytes and their byproducts triggers microglial cell activation, which in turn leads to the release of inflammatory cytokines, initiating a potent inflammatory cascade [4]. This cascade significantly harms neural tissues, resulting in neurobehavioral changes, cerebral edema, and compromised blood-brain barrier integrity [5–7]. After ICH, the weakened blood-brain barrier allows neurogenic inflammatory cytokines to enter systemic circulation, potentially triggering a chain of inflammatory responses, leading to systemic inflammatory response syndrome and even multi-organ dysfunction [2, 8]. Therefore, elevated cytokine levels could indicate central nervous system (CNS) deterioration following ICH.

Historically, the complex pathophysiology of ICH has been thoroughly investigated using experimental animal models, with neuroinflammation standing out as a key area of therapeutic interest [9, 10]. The neuroinflammatory cascade, triggered by ICH, is thought to be a critical physiological response to neural trauma [6]. In cases of ICH, the infiltration of neural tissue by blood components activates both innate and adaptive immune responses, attracting peripheral blood leukocytes to areas near hematomas [11, 12]. This results in the release of crucial inflammatory mediators, such as tumor necrosis factor-α (TNF-α), interleukins (IL), and chemokines, which play a significant role in acute inflammatory responses following neural injury [4]. Numerous previous clinical studies have highlighted increased levels of these inflammatory markers in the serum of ICH patients, linking them to neurological deficits, cerebral edema, and poor clinical outcomes [13–16]. Since cerebrospinal fluid (CSF) closely interacts with neural tissues, monitoring changes in CSF inflammatory cytokine levels, especially their movement from neural tissues into CSF, offers critical insight into neuroinflammatory processes, reflecting the stages of neural or neuronal injury [17–19].

Therefore, the primary aim of this study is to closely examine the relationship between changes in CSF inflammatory cytokine levels in ICH patients and the progression of ICH, as well as their association with the severity of neural damage and neurological prognosis. The ultimate goal is to use these findings to improve diagnostic accuracy and treatment strategies for ICH.

## Materials and methods

### Participants

This study included 100 patients diagnosed with new-onset ICH, admitted to the General Hospital between September 2019 and January 2023, as the primary cohort. **Inclusion Criteria for ICH Patients**: (1) Individuals presenting with primary cerebral hemorrhage admitted within 24 h of symptom onset; (2) Patients meeting strict diagnostic criteria for SICH, confirmed through comprehensive clinical evaluations, including medical history, neurological examinations, and imaging diagnostics [20]. **Exclusion Criteria**: (1) Patients with acute hemorrhage due to ruptured vascular malformations, aneurysms, or hemorrhage related to neoplastic growths or trauma; (2) Individuals diagnosed with malignant neoplasms; (3) Patients with significant organ dysfunction, including cardiac, hepatic, and renal impairments; (4) Those with a cerebrovascular event in the past three months; (5) Conditions like congenital vascular malformations, vasculitis, and autoimmune diseases; (6) Recent use of oral anticoagulant or antiplatelet medications; (7) Patients with ICH requiring an external ventricular drain (EVD) for obstructive hydrocephalus management; (8) Patients with contraindications to or unwillingness for CSF extraction. A control group of 50 patients without CNS disorders, treated during the same period, was also included. These patients underwent CSF sampling due to symptoms such as fever, headache, nausea, vomiting, and neck stiffness, leading to suspicions of a CNS disease. **Inclusion Criteria for Control Group**: (1) No CNS pathologies (including cerebrovascular conditions, CNS infections, demyelinating and degenerative disorders), confirmed by cranial CT/MRI; (2) Normal findings in routine CSF diagnostics and biochemistry; (3) No history of cardiovascular or cerebrovascular diseases. Detailed demographic, clinical, and laboratory data were collected from both participant groups. The study protocol was approved by the Ethics Committee of the General Hospital of Xinjiang Military Command, reference number 2023RR0301. Informed consent was obtained from all patients; for unconscious patients, their close relatives were informed, and their consent was acquired in writing.

### CSF acquisition

CSF samples were collected via lumbar puncture and immediately subjected to clinically required diagnostic tests upon admission. For this study, CSF samples were gathered within 48 h of initial hospital admission, with the average time from hospitalization to sampling for ICH patients being 18 ± 6 h. During collection and evaluation, we strictly followed the Standard Operating Procedures (SOPs). After collection, CSF aliquots were centrifuged at 3,000 RPM for 10 min at a controlled temperature of 4 °C, then cryogenically stored at -80 °C.

### Inflammatory cytokine detection

Initially, CSF samples from 27 ICH patients and 15 controls were analyzed to quantify the expression of various cytokines: IL-1β, IL-2, IL-4, IL-6, IL-8, IL-10, IL-12P70, IL-13, TNF-α, and interferon (INF)-γ, using the Mesoscale Discovery Platform (MSD, Rockville, MD, USA) electrochemiluminescence method. MSD is advantageous due to its minimal sample volume requirements and capability for rapid multiplex analysis. The MSD technology relies on creating a two-part antibody sandwich complex, consisting of a capture antibody and a detection antibody tagged with ruthenium tricyclic. When electrically stimulated, the ruthenium tricyclic emits light in a chemiluminescent reaction, with the intensity of emitted photons linearly correlating with the concentration of the target analyte. Observing notable increases in inflammatory cytokines in ICH patients compared to controls during preliminary MSD screenings, we expanded the sample size for further validation. CSF samples from 100 ICH patients and 50 controls were then precisely assayed using specific enzyme-linked immunosorbent assay (ELISA) kits for IL-6 (Ref: E-EL-H6156), IL-8 (Ref: E-EL-H6008), IL-10 (Ref: E-EL-H6154), and TNF-α (Ref: E-EL-H0109c), obtained from Wuhan Elite Bioscience and Technology Co, Ltd. These assays were performed strictly according to the manufacturer’s instructions. The absorbance at 450 nm (OD450) was measured following colorimetric reactions, allowing for the calculation of target cytokine concentrations using standard curve methodology.

### Judgement criteria of disease severity

The assessment of disease severity in ICH patients upon admission was based on the Glasgow Coma Scale (GCS) [21]. Scores ranging from 9 to 15 were considered indicative of mild to moderate disease, while scores from 3 to 8 were classified as severe disease presentations.

### Diagnosis of acute hydrocephalus

Cerebral CT scans were systematically performed on patients diagnosed with ICH. The diagnosis of hydrocephalus was based on the presence of any of these radiographic indicators: (1) clear dilation of the temporal horn, (2) significant bulging of the third ventricular wall, (3) rounding of the frontal horn, (4) effacement of cerebral sulci, and (5) ventricular enlargement disproportionate to sulcal dilation. The identification of any of these features in the cerebral CT scan confirmed the diagnosis of acute hydrocephalus.

### Prognostic judgement criteria

A six-month post-discharge longitudinal follow-up was conducted for ICH patients. Prognostic outcomes were evaluated using the Glasgow Outcome Scale (GOS) [22]. A GOS score of 4 to 5 (where 4 indicates mild disability and 5 denotes good recovery) was interpreted as a favorable prognosis. In contrast, a GOS score of 1 to 3 (with 1 representing death, 2 a vegetative state, and 3 severe disability) was considered indicative of a poor prognosis.

### Data statistics

For data analysis, we used software platforms SPSS (Version 26.0) and GraphPad Prism (Version 8.0.2). Categorical variables were presented as case counts, and intergroup comparisons were conducted using the χ2 test. Quantitative data following a normal distribution were expressed as mean ± standard deviation, with the t-test applied for comparing continuous variables between two groups. Non-normally distributed datasets were represented as median with interquartile range (M [P25, P75]), and comparisons between groups were performed using the Mann-Whitney U test. Correlations were examined using Spearman’s rank correlation coefficient. Receiver Operating Characteristic (ROC) curves were generated to assess the predictive value of cytokine levels (specifically IL-6, IL-8, IL-10, and TNF-α) for prognosis in the ICH cohort. A *p*-value of less than 0.05 was considered statistically significant.

## Results

### Clinical data of study subjects

The study involved 100 patients with ICH and 50 control individuals, selected based on the defined inclusion criteria. The ICH group comprised 65 males and 35 females, with an average age of 58.52 ± 13.22 years. The median GCS score for this cohort was 9 (IQR, 5–12). The control group consisted of 36 males and 14 females, with an average age of 31.02 ± 15.00 years. A detailed comparison of demographic and clinical parameters is provided in Table 1.

**Table 1:** Demographic and Baseline Characteristics

Characteristic	Control group (n = 50)	ICH group (n = 100)	*P* value
Sex, n (%)			0.389
Female	14(28)	35(35)	
Male	36(72)	65(65)	
Age, yr, mean (SD)	31.02(15.00)	58.52(13.22)	< 0.0001*
BMI(Kg/m2), median (IQR)	24.22(23.39–26.23)	24.22(23.43–26.03)	0.484
Hypertension, n (%)	12(24)	55(55)	< 0.001*
Diabetes, *n* (%)	4(8)	12(12)	0.454
Hematoma location, *n* (%)			N/A
Basal ganglia	N/A	45(45)	
Lobar	N/A	14(14)	
Thalamus	N/A	26(26)	
Cerebellum	N/A	6(6)	
Brainstem	N/A	3(3)	
Ventricle	N/A	6(6)	
Into the ventricle, n (%)	N/A	45(45)	N/A
Final ICH volume(ml), n (%)			N/A
< 30	N/A	52(52)	
≥ 30	N/A	48(48)	
Hydrocephalus, n (%)	N/A	16(16)	N/A
GCS, median (IQR)	N/A	9(5–12)	N/A
Time from symptom onset to MIS (hours), mean (SD)	N/A	4.15(3.62)	N/A
Infection while in hospital, *n* (%)	N/A	27(27)	N/A
Routine examination of CSF			
WBC(n), median (IQR)	3(2–6)	20(6-233)	< 0.0001*
RBC(n), median (IQR)	6(3.75-10)	3580(212.5-13100)	< 0.0001*
Biochemical reactions of CSF			
PRO(g/L), median (IQR)	0.245(0.190–0.310)	1.10(0.53–2.20)	< 0.0001*
GLU (mmol/L), median (IQR)	3.315(3.133–3.603)	3.830(3.180–5.640)	< 0.001*
CL (mmol/L), median (IQR)	121.4(120.4-122.8)	122.5(117.6-127.3)	0.471
GOS, n (%)			N/A
1 ~ 3	N/A	53(53)	
4 ~ 5	N/A	47(47)	

Note: The primary disease or clinical reason/indication for CSF sampling in the control group was due to the patients displaying symptoms such as fever, headache, nausea, vomiting, and neck stiffness. These symptoms led clinicians to suspect the presence of a central nervous system disease

Abbreviations: GCS, Glasgow coma scale; ICH, Intracerebral hemorrhage; IQR, Interquartile range; MIS, minimally invasive surgery; SD, standard deviation; BMI, Body Mass Index; CSF, Cerebrospinal fluid; WBC, White blood cell; RBC, Red blood cell; PRO, protein; GLU, glucose; GOS, Glasgow outcome scale. * Statistically significant

### Elevated levels of inflammatory cytokine expression in CSF of ICH patients

**Fig. 1:**
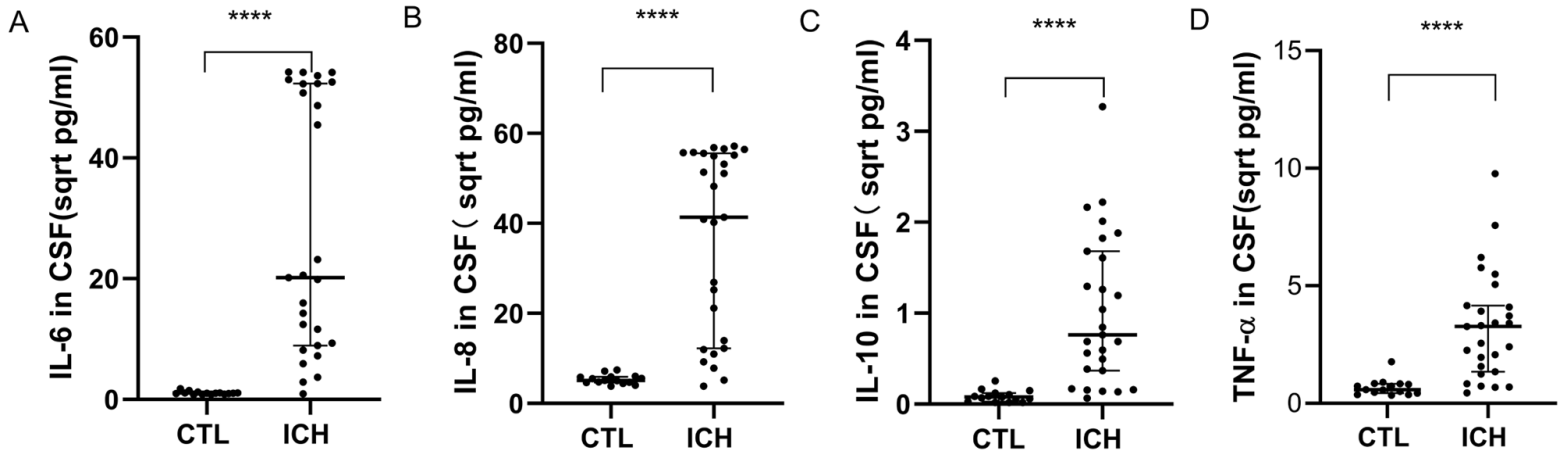
Discrepancies in Expression Levels of IL-6, IL-8, IL-10, and TNF-α in the CSF of ICH Patients and Controls as Unearthed by Preliminary Assessment. Utilizing the MSD methodology, expression levels of 10 inflammatory cytokines in the CSF of both ICH patients and controls were meticulously evaluated. It was discerned that the concentrations of (**A**) IL-6 (*P* < 0.0001), (**B**) IL-8 (*P* < 0.0001), (**C**) IL-10 (*P* < 0.0001), and (**D**) TNF-α (*P* < 0.0001) were markedly elevated in comparison to controls, whilst the remaining cytokines did not exhibit significant differential expression (data not depicted). Note: The original result values have been transformed to their square root equivalents to facilitate a more distinct and three-dimensional portrayal of data distribution, denoted in sqrt pg/ml. Data are delineated as M (P25, P75) with **** indicating *P* < 0.0001. Abbreviations: CTL, control, n = 15; ICH, intracerebral hemorrhage, n = 27

The CSF of ICH patients was examined for inflammatory cytokines using the MSD electrochemiluminescence method. This analysis revealed significantly higher concentrations of IL-6, IL-8, IL-10, and TNF-α in ICH patients compared to controls (*P* < 0.05) (Fig. 1A-D). Cytokines without statistically significant differences were excluded from this presentation. The initial smaller sample size for this preliminary screening might introduce potential biases, emphasizing the need for validation with a larger sample size.

**Fig. 2:**
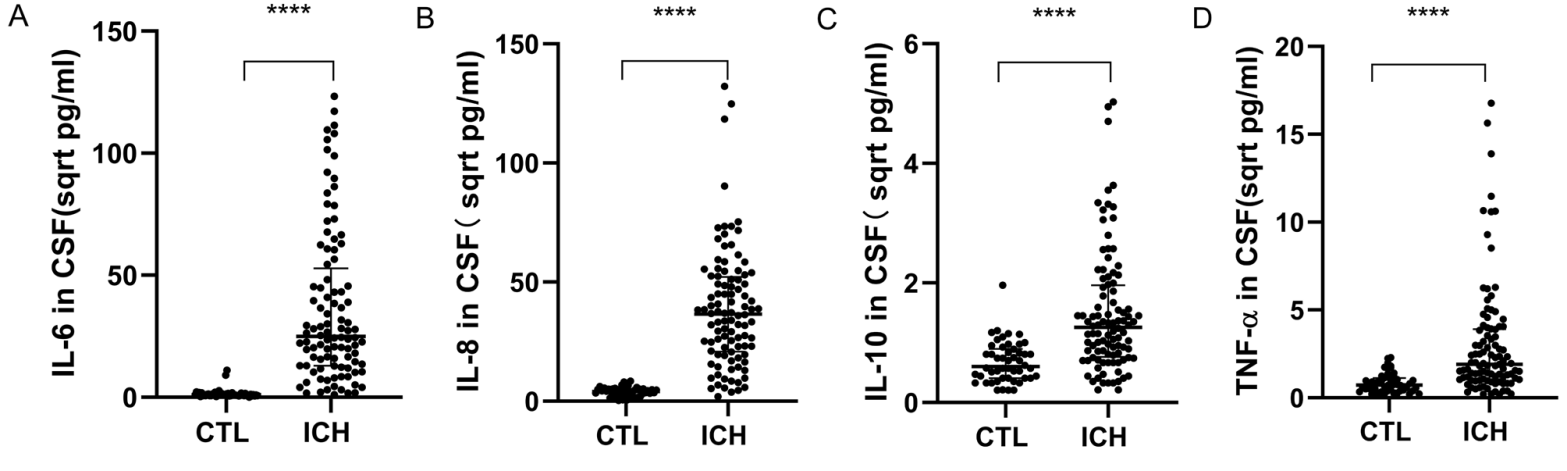
Validation Study Employing Expanded Sample Sizes Underscores Disparities in IL-6, IL-8, IL-10, and TNF-α Expression Levels in the CSF between ICH Patients and Controls. Expression dynamics of IL-6, IL-8, IL-10, and TNF-α in the CSF, as determined via ELISA, revealed that (**A**) IL-6 (*P* < 0.0001); (**B**) IL-8 (*P* < 0.0001); (**C**) IL-10 (*P* < 0.0001); (**D**) TNF-α (*P* < 0.0001) were substantially heightened in the ICH cohort. Note: The original result values have been transformed to their square root equivalents to facilitate a more distinct and three-dimensional portrayal of data distribution, denoted in sqrt pg/ml. Data are encapsulated as M (P25, P75) with **** denoting *P* < 0.0001. Abbreviations: CTL, control, n = 50; ICH, intracerebral hemorrhage, n = 100

Consequently, the sample sizes for both ICH patients and controls were increased for a more detailed examination of inflammatory cytokine levels in the CSF using ELISA. Compared to the control group, ICH patients showed significantly higher levels of IL-6, IL-8, IL-10, and TNF-α in their CSF, each with statistical significance (*P* < 0.05) (Fig. 2A-D).

### Disease severity of ICH correlates with inflammatory cytokine levels in the CSF

**Fig. 3:**
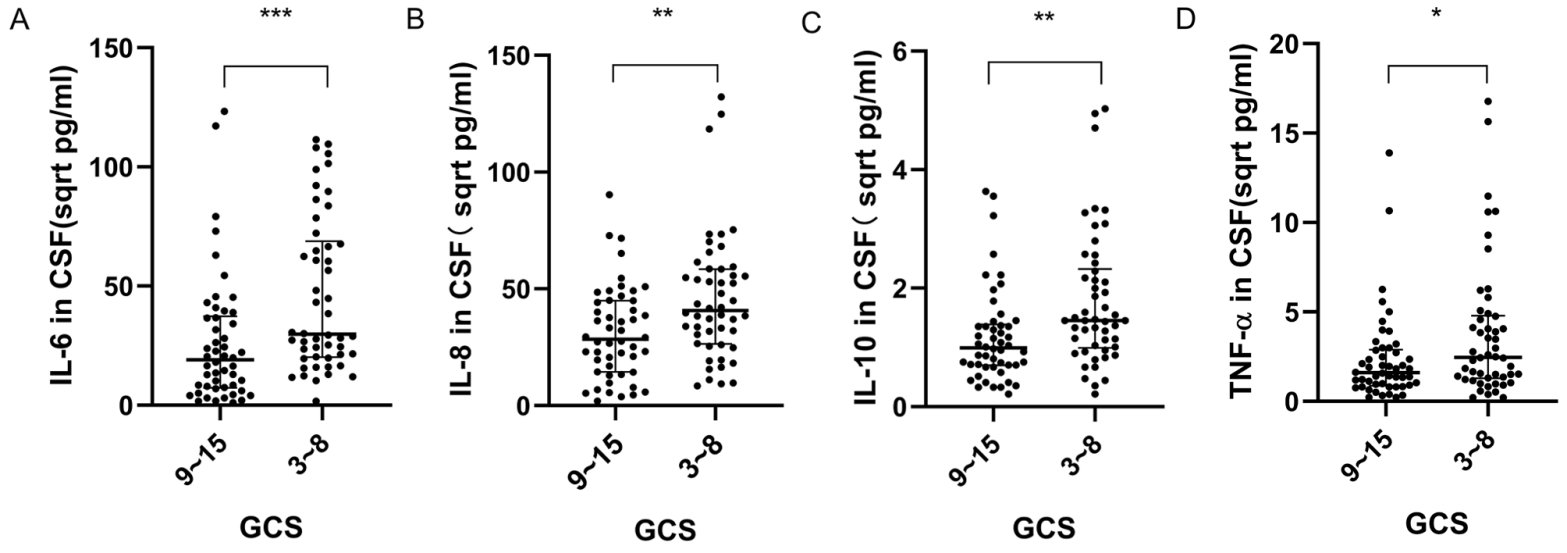
Variability in Expression Levels of IL-6, IL-8, IL-10, and TNF-α in CSF Among ICH Patients Stratified by GCS Values. (**A**) IL-6 concentrations in the CSF of ICH patients possessing a GCS between 3–8 were notably elevated when juxtaposed to those encompassing a GCS of 9–15 (*p* < 0.001). (**B**) IL-8 concentrations were discernibly enhanced in the CSF of ICH patients within the 3–8 GCS range compared to those in the 9–15 range (*p* < 0.01). (**C**) ICH patients with a GCS range of 3–8 displayed pronounced IL-10 levels in the CSF relative to the 9–15 GCS cohort (*p* < 0.01). (**D**) CSF TNF-α concentrations in ICH patients situated in the 3–8 GCS range exceeded those in the 9–15 GCS cohort (*p* < 0.05). Note: The original result values have been transformed to their square root equivalents to facilitate a more distinct and three-dimensional portrayal of data distribution, denoted in sqrt pg/ml. Data is tabulated as M (P25, P75), with *** indicating *P* < 0.001, ** denoting *P* < 0.01, and * representing *P* < 0.05. Both GCS cohorts, 3–8 and 9–15, consisted of n = 50

**Fig. 4:**
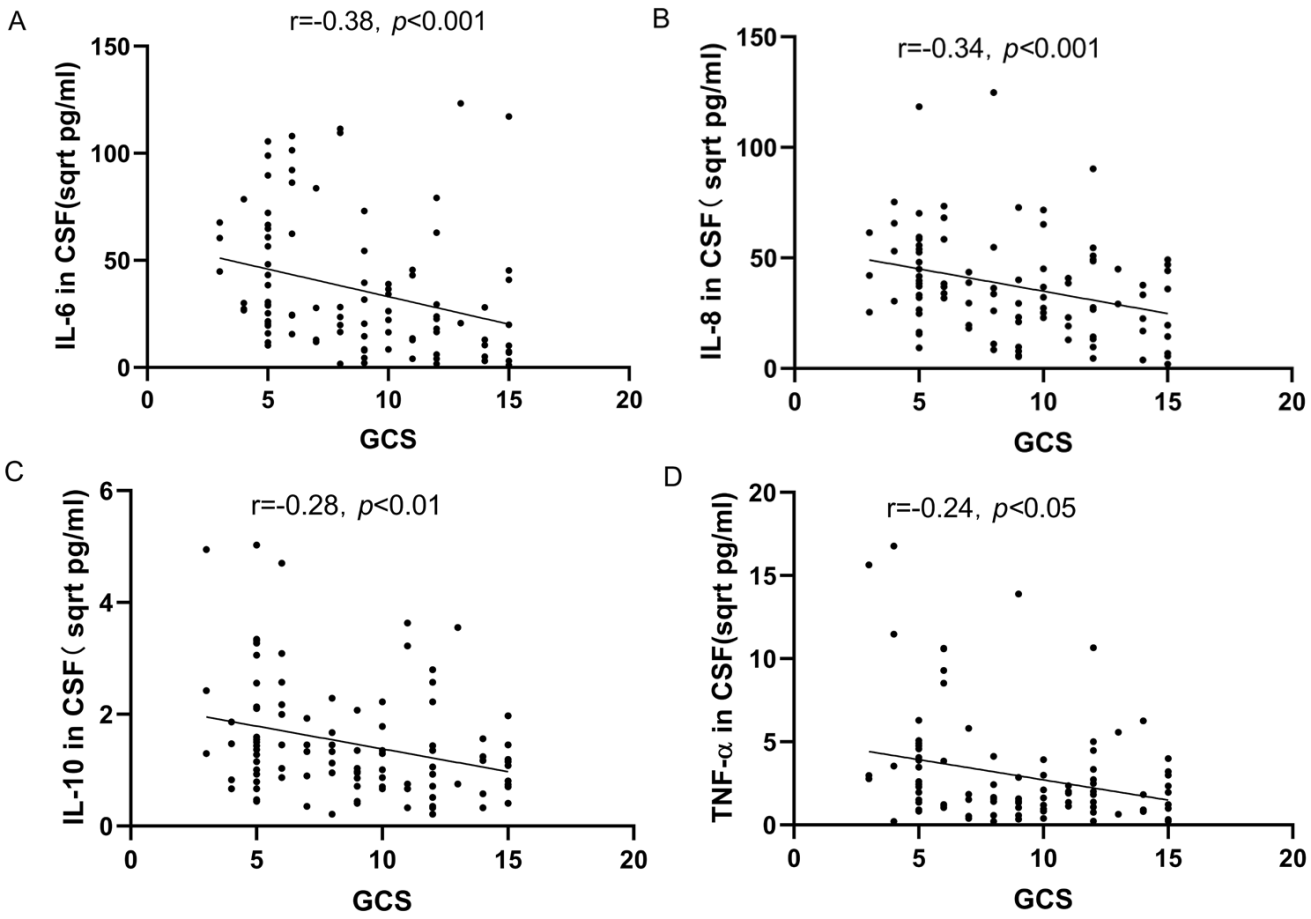
Correlative Analysis of IL-6, IL-8, IL-10, and TNF-α Expression Levels in CSF with Disease Severity in ICH Patients. (**A**) An inverse relationship was noted between IL-6 levels and GCS, with a correlation coefficient of r=-0.38 (*p* < 0.001). (**B**) IL-8 concentrations showcased a negative affiliation with GCS (r=-0.34, *p* < 0.001). (**C**) A reverse correlation between IL-10 levels and GCS was determined (r=-0.28, *p* < 0.01). (**D**) TNF-α levels in CSF were found to be inversely proportional to GCS (r=-0.24, *p* < 0.05). Note: The original result values have been transformed to their square root equivalents to facilitate a more distinct and three-dimensional portrayal of data distribution, denoted in sqrt pg/ml. The data set is structured as M (P25, P75). Both GCS brackets, 3–8 and 9–15, were represented by n = 50

In the study, higher levels of IL-6, IL-8, IL-10, and TNF-α were observed in the CSF of patients with GCS scores between 3 and 8 compared to those with scores ranging from 9 to 15 (*P* < 0.05) (Fig. 3A-D). Further analysis was conducted to explore the relationship between the severity of ICH and inflammatory cytokine levels in the CSF. This investigation revealed that CSF levels of IL-6, IL-8, IL-10, and TNF-α were inversely correlated with GCS scores (*r*=-0.38, *p* < 0.001; *r*=-0.34, *p* < 0.001; *r*=-0.28, *p* < 0.01; *r*=-0.24, *p* < 0.05). This suggests that a decrease in clinical severity, as indicated by higher GCS scores, is associated with lower levels of these inflammatory cytokines in the CSF (Fig. 4A-D).

### Higher levels of inflammatory cytokines in CSF of patients with hydrocephalus

**Fig. 5:**
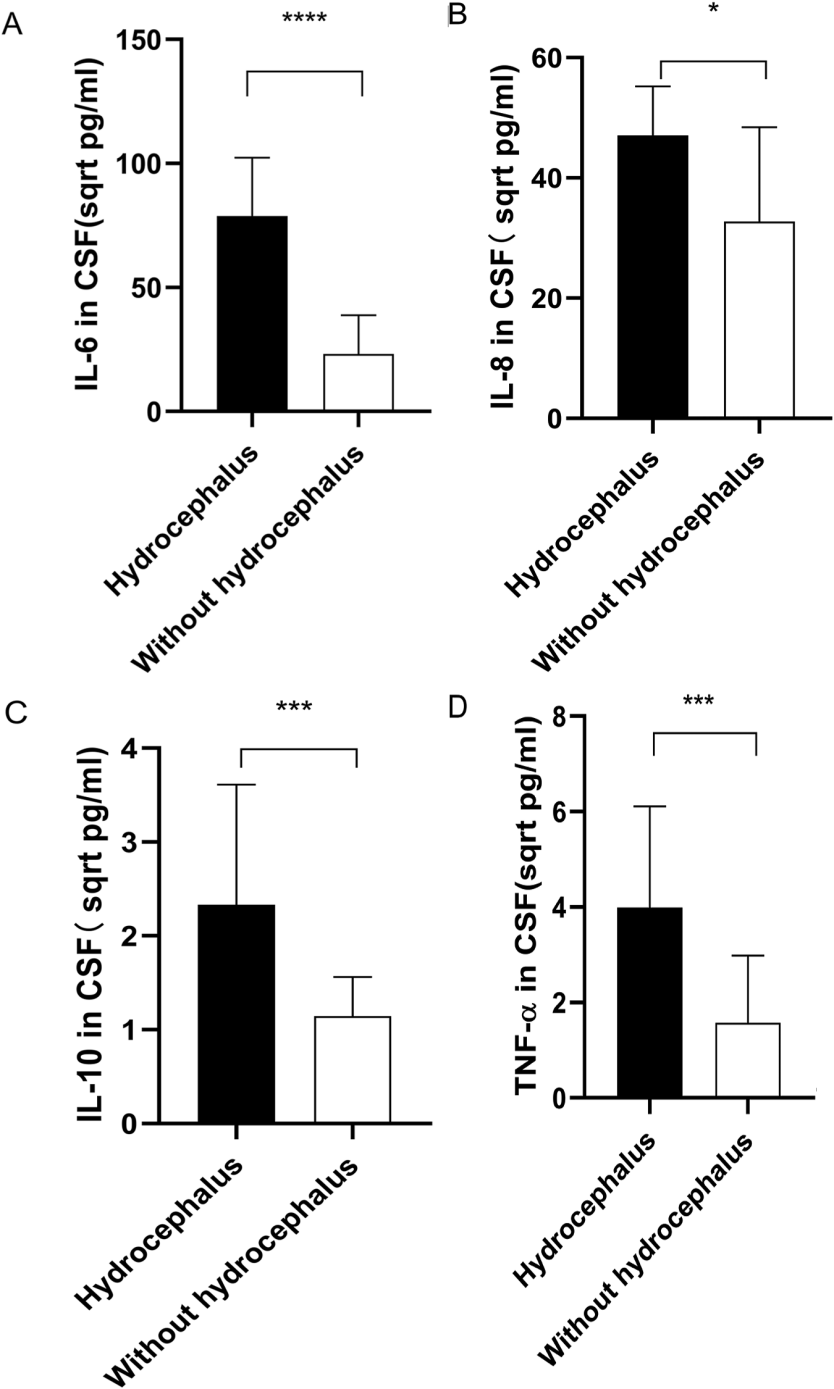
Differential Cytokine Expression Profiles in the CSF Among ICH Patients: A Comparison between Hydrocephalic and Non-hydrocephalic Subjects. (**A**) IL-6 concentrations in the CSF of hydrocephalic ICH patients were significantly elevated (*P* < 0.0001). (**B**) IL-8 levels were augmented (*P* < 0.05). (**C**) IL-10 (*P* < 0.001) and (**D**) TNF-α (*P* < 0.001) levels were also observed to be higher in patients with hydrocephalus relative to their non-hydrocephalic counterparts. Note: The original result values have been transformed to their square root equivalents to facilitate a more distinct and three-dimensional portrayal of data distribution, denoted in sqrt pg/ml. The dataset is delineated as M (P25, P75). Annotations: * for *P* < 0.05, *** for *P* < 0.001, **** for *P* < 0.0001. The hydrocephalus cohort comprised n = 16; whereas the non-hydrocephalic cohort consisted of n = 84

Patients diagnosed with both ICH and hydrocephalus exhibited significantly higher concentrations of IL-6, IL-8, IL-10, and TNF-α in their CSF compared to those without hydrocephalus (*P* < 0.0001, *P* < 0.05, *P* < 0.001, and *P* < 0.001 respectively), as depicted in Fig. 5A-D.

### Relationship between inflammatory cytokine expression levels in CSF of ICH patients and prognosis of ICH

**Fig. 6:**
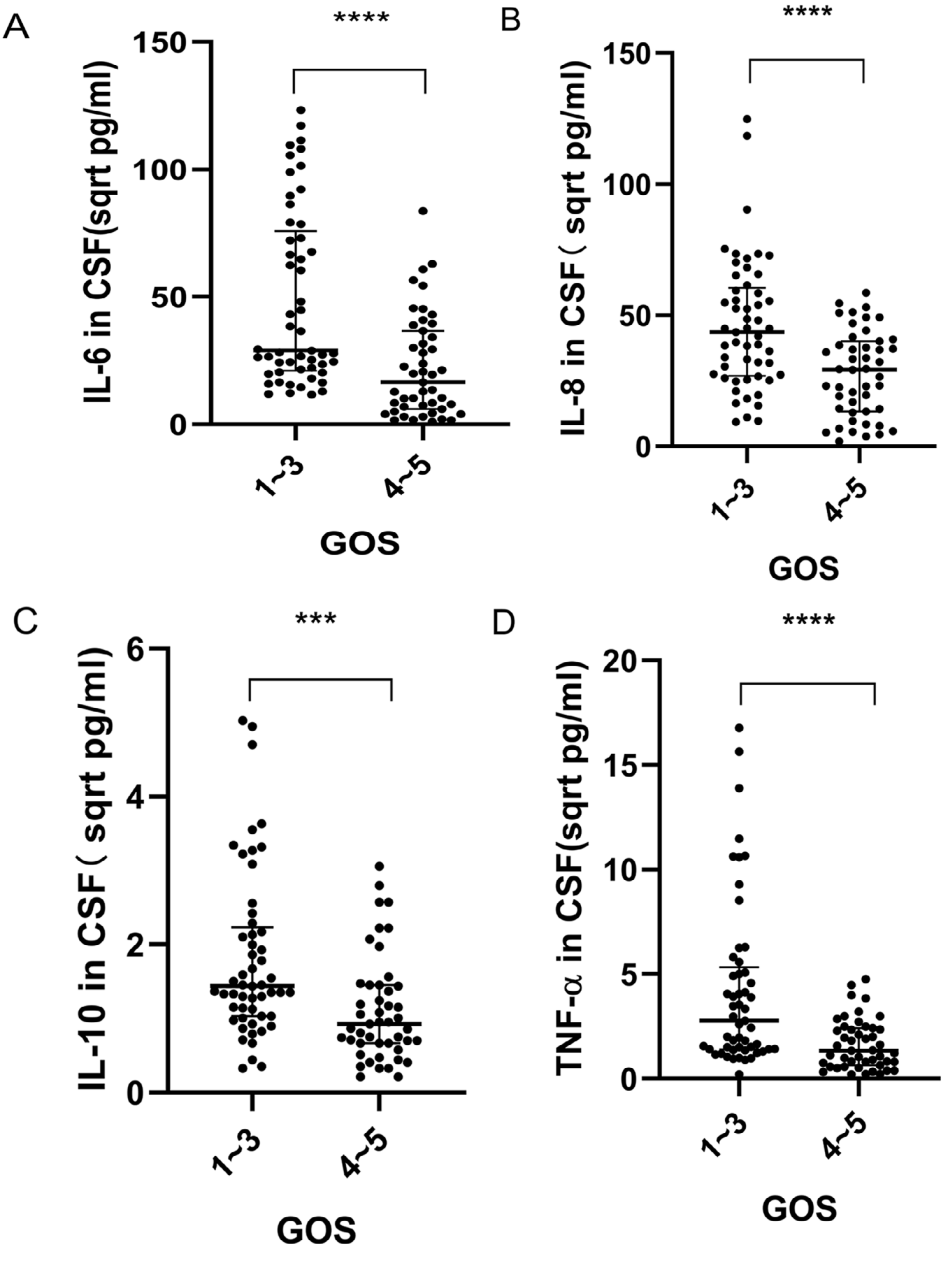
Comparative Analysis of IL-6, IL-8, IL-10, and TNF-α CSF Expression Across ICH Patients Stratified by GOS. (**A**) CSF concentrations of IL-6 in subjects with GOS scores between 1 and 3 notably exceeded those with scores between 4 and 5 (*p* < 0.0001). (**B**) Similarly, IL-8 CSF concentrations for GOS scores of 1 to 3 were elevated in comparison to scores of 4 to 5 (*p* < 0.0001). (**C**) IL-10 levels in the CSF were elevated for GOS scores of 1 to 3 relative to 4 to 5 (*p* < 0.001). (**D**) TNF-α concentrations within the CSF of subjects with GOS scores of 1 to 3 markedly surpassed those scoring between 4 and 5 (*p* < 0.0001). Note: The original result values have been transformed to their square root equivalents to facilitate a more distinct and three-dimensional portrayal of data distribution, denoted in sqrt pg/ml. Data is systematically presented as M (P25, P75), with *** denoting *P* < 0.001, and **** indicating *P* < 0.0001. Cohorts: GOS of 1–3, n = 53; GOS of 4–5, n = 47

**Fig. 7:**
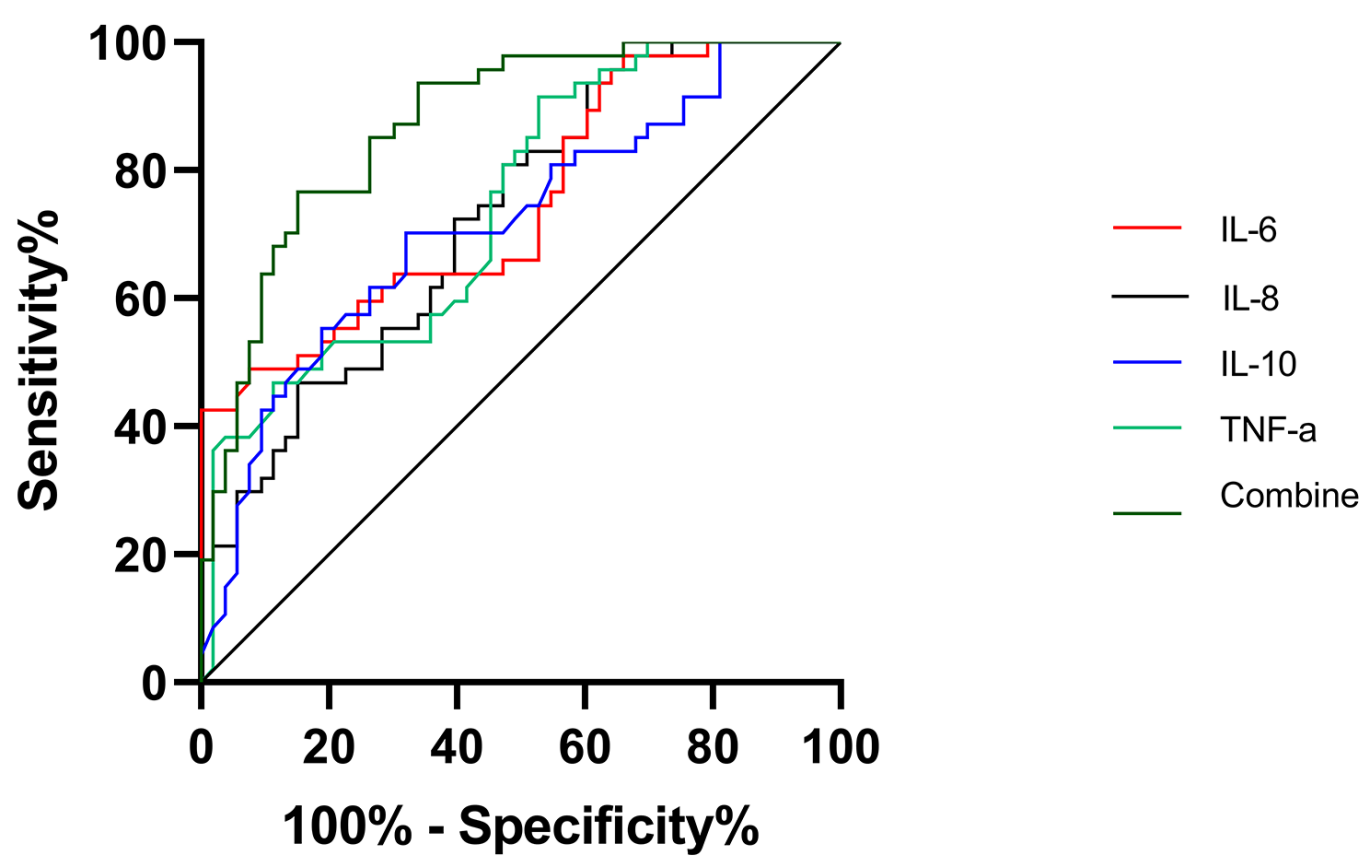
Predictive Capability Evaluation of IL-6, IL-8, IL-10, and TNF-α CSF Expression Levels in ICH Patients, and the Synergetic Prognostic Value of the Composite. The graphical representations are color-coded with red representing IL-6, black signifying IL-8, blue indicative of IL-10, green denoting TNF-α, and dark green symbolizing the amalgamated set, with a total sample size of n = 100

Over a 6-month period, patients were followed and subsequently categorized based on their prognosis: those with a favorable prognosis (GOS, between 4 and 5) and those with an unfavorable prognosis (GOS ranging from 1 to 3). Initial univariate analysis revealed that several factors, including age, hemorrhagic volume, coexisting conditions, GCS score, total protein, and glucose levels in CSF, significantly affected the prognostic outcomes of ICH (Table 2). Concentrations of IL-6, IL-8, IL-10, and TNF-α in the CSF were higher in patients with poor prognoses compared to those with favorable prognoses (Fig. 6A-D). Logistic regression analysis was conducted to identify the factors most predictive of a poor prognosis. It was found that GCS score and CSF levels of IL-6, IL-8, IL-10, and TNF-α were key determinants of prognosis (Table 3). ROC analysis showed that the area under the curve (AUC) for the inflammatory markers IL-6, IL-8, IL-10, TNF-α, and their combined predictive ability for ICH prognosis in CSF was 0.750, 0.728, 0.728, 0.717, 0.743, and 0.874, respectively, with sensitivities of 92.5%, 52.8%, 67.9%, 47.2%, and 84.9%, and specificities of 48.9%, 80.9%, 70.2%, 91.5%, and 76.6%, respectively, as shown in Fig. 7 and detailed in Table 4.

**Table 2:** Univariate analysis for the clinical characteristics of subjects with a favorable or unfavorable outcome

Characteristic	Favorable (n = 47)	Unfavorable (n = 53)	*P* value
Sex, n (%)			0.515
Female	18(38.3)	17(32.1)	
Male	29(61.7)	36(67.9)	
Age, yr, mean (SD)	55.28(13.67)	61.40(12.22)	< 0.05*
BMI(Kg/m2), median (IQR)	24.22(22.49–26.45)	24.00(23.43–26.10)	0.966
Hypertension, n (%)	21(44.7)	34(64.2)	< 0.05*
Diabetes, *n* (%)	1(2.1)	11(20.8)	< 0.01*
Hematoma location, *n* (%)			0.248
Basal ganglia	17(36.2)	28(52.8)	
Lobar	9(19.1)	5(9.4)	
Thalamus	12(25.5)	14(26.4)	
Cerebellum	5(10.6)	1(1.8)	
Brainstem	1(2.1)	2(3.8)	
Ventricle	3(6.4)	3(5.7)	
Into the ventricle, n (%)	17(36.2)	28(52.8)	0.095
Final ICH volume(ml), n (%)			< 0.001*
< 30	34(72.3)	18(34)	
≥ 30	13(27.7)	35(66)	
Hydrocephalus, n (%)	6(12.8)	10(18.9)	0.406
GCS, median (IQR)	11(9–14)	6(5–8)	< 0.0001*
Time from symptom onset to MIS (hours), mean (SD)	4.091(4.302)	4.235(2.547)	0.176
Infection while in hospital, *n* (%)	10(21.3)	17(32.1)	0.225
Routine examination of CSF			
WBC(n), median (IQR)	3040(160–9190)	4280(225-18540)	0.213
RBC(n), median (IQR)	8(4-120)	80(6-420)	0.073
Biochemical reactions of CSF			
PRO(g/L), median (IQR)	0.78(0.44–1.44)	1.64(0.6-2.645)	< 0.01*
GLU (mmol/L), median (IQR)	3.47(3.080–4.88)	4.605(3.3-6.323)	< 0.05*
CL (mmol/L), median (IQR)	121.8(117.1-125.6)	122.9(118.1-131.5)	0.134

Abbreviations: GCS, Glasgow coma scale; ICH, Intracerebral hemorrhage; IQR, Interquartile range; MIS, minimally invasive surgery; SD, standard deviation;BMI, Body Mass Index; CSF, Cerebrospinal fluid; WBC, White blood cell; RBC, Red blood cell; PRO, protein; GLU, glucose. * Statistically significant

**Table 3:** Logistic regression analysis parameters affecting prognosis of ICH patients

Variables	b	*P*	OR	95%CI
IL-6	0.001	0.001	1.001	1.000-1.001
IL-8	0.001	0.000	1.001	1.000-1.001
IL-10	0.218	0.009	1.244	1.056–1.466
TNF-α	0.134	0.001	1.143	1.055–1.239
GCS	-0.437	0.000	0.646	0.546–0.764

Abbreviations: b, coefficient; GCS, Glasgow coma scale

**Table 4:** The expression levels of IL-6, IL-8, IL-10 and TNF-α in CSF and the efficacy indexes of the four factors in predicting ICH prognosis

Cytokines	AUC	Sensitivity (%)	Specificity (%)	95%CI	Youden index	Cutoff value (pg/ml)	*p*
IL-6	0.750	92.5	48.9	0.654–0.845	0.414	198.01	< 0.0001
IL-8	0.728	52.8	80.9	0.632–0.825	0.337	1756.64	< 0.0001
IL-10	0.717	67.9	70.2	0.616–0.818	0.318	1.58	< 0.001
TNF-α	0.743	47.2	91.5	0.648–0.839	0.387	10.72	< 0.0001
Combine	0.874	84.9	76.6	0.806–0.941	0.615	—	< 0.0001

## Discussion

In the current healthcare setting, managing ICH presents a significant challenge, with high associated mortality and morbidity rates [23]. It is crucial to accurately assess neurological impairment and predict outcomes in ICH patients at early stages to enable prompt and effective interventions. These interventions are key to reducing morbidity, mortality, and disability in these patients. ICH triggers a complex sequence of pathophysiological responses. The presence of blood within the brain initiates an inflammatory response, which is fundamental to both immediate and long-term cerebral damage following ICH. This process suggests a potential therapeutic target [19, 24]. Past animal studies and clinical research have emphasized the importance of post-ICH inflammatory reactions in recovering neurological function and determining the overall prognosis of ICH. Inflammatory cytokines are central in the neuroinflammatory aftermath of ICH, with cytokines like IL-6, IL-10, and TNF-α playing key roles in modulating immune responses and central nervous system functions [13, 25–27].

Our study identified a significant increase in CSF levels of inflammatory cytokines IL-6, IL-8, IL-10, and TNF-α upon initial admission in patients with ICH. In contrast, cytokines such as IL-1β, IL-2, IL-4, IL-12P70, IL-13, and INF-γ did not show significant differences. Contemporary research suggests that aseptic inflammatory reactions are common in patients experiencing ventricular hemorrhage following the hemorrhagic event. A heightened inflammatory response correlates with the initial hemorrhage size, as evidenced by an increased leukocyte count in CSF adjusted for hemorrhage [17, 28]. This aligns with the trends of inflammatory cytokines observed in our study. Similar findings of increased CSF inflammatory cytokine levels, including IL-1α, IL-1β, IL-2, IL-4, IL-6, INF-γ, and TNF-α, have been reported in cases of aneurysmal subarachnoid hemorrhage [29–31]. Huang et al. [32] highlighted that elevated CSF S100B levels in ICH patients could enhance IL-1β expression in primary microglia through ERK1/2, JNK, and p38 MAPK pathways. IL-1β, a pro-inflammatory cytokine, initiates an acute-phase reaction in the CNS, suggesting a role for the cerebral injury biomarker S100B in influencing inflammatory dynamics after cerebral hemorrhage. Similarly, Wendy et al. [33] observed increased CSF levels of IL-1β, IL-6, IL-10, TNF-α, and CCL2 following ventricular hemorrhage, which later decreased in subsequent assessments. Elevated IL-8 levels in the CSF of neonates with ventricular hemorrhage have also been reported [34, 35], with animal model studies corroborating these findings [36–38].

These studies collectively confirm the occurrence of a neuroinflammatory cascade in brain tissues following ICH. However, the specific cytokine profile found in CSF reflecting this inflammation does not entirely align with our findings. This discrepancy may be due to variations in hemorrhage location, volume, and disease progression among the subjects studied, indicating a need for more detailed research.

After an ICH, there is a strong correlation between the levels of inflammatory cytokines in the CSF and the degree of cerebral tissue damage. Wendy et al. [33] reported a significant relationship between CSF concentrations of IL-6, IL-8, and IL-10, and the volume of cerebral hemorrhage. Furthermore, a clear link was observed between the admission GCS score and the levels of TNF-α and IL-8. In a similar context, Yan et al. [39] found that levels of TNF-α, IL-6, and IL-1β in the blood or CSF of patients with acute spontaneous ICH were significantly higher compared to patients with mild ICH or healthy individuals. Additionally, Belarbi et al. [40] noted that increased levels of IL-11, TNF-α, and vascular endothelial growth factor correlated with higher neurologic deficit scores, indicating more severe neurological damage.

In our study, ICH patients with lower GCS scores had higher CSF concentrations of IL-6, IL-8, IL-10, and TNF-α. Further correlation analysis reinforced that the levels of IL-6, IL-8, IL-10, and TNF-α were inversely related to the GCS. This finding aligns with, but is not entirely consistent with, previous literature. Since CSF is an extracellular fluid of the CNS, analyzing it offers critical insights into the condition of the CNS. These findings highlight the potential of using CSF inflammatory cytokine levels as a marker for cerebral damage after ICH, providing a valuable tool for assessing the status of ICH patients.

Hydrocephalus is a common complication following intraventricular and subarachnoid hemorrhages. Katherine et al. [41] explored the neuroinflammatory mechanisms underlying hydrocephalus development after intraventricular hemorrhage and concluded that inflammatory cytokines play a key role in this process. Similarly, Gakwaya et al. [42] found that cytokine levels, specifically TNF-α, IL-1α, IL-4, IL-6, and IL-12, were significantly higher in the CSF of neonates with posthemorrhagic hydrocephalus. It is important to note that the CNS of preterm neonates is still developing, which may lead to differences in response compared to adults.

In our study, we investigated the relationship between hydrocephalus and CSF inflammatory cytokine levels in patients with ICH. We observed that ICH patients with hydrocephalus had higher levels of IL-6, IL-8, IL-10, and TNF-α in their CSF compared to those without hydrocephalus. This suggests that cerebral hemorrhages, especially when accompanied by hydrocephalus, amplify the neuroinflammatory response, leading to increased cerebral tissue damage and potentially affecting the patient’s prognosis. This finding underscores the impact of hydrocephalus on the inflammatory response in the brain and its significance in the clinical management of ICH patients.

The relationship between the neuroinflammatory response and prognosis following ICH remains somewhat unclear. While some studies on spontaneous intraventricular hemorrhage reported no significant link between aseptic inflammation in the CSF and poor prognosis [17, 18], another study [39] indicated that ICH patients with elevated levels of TNF-α in blood or CSF had a slightly increased risk of adverse outcomes. This study also found that patients with higher IL-6 levels were much more likely to experience unfavorable outcomes, suggesting the importance of measuring TNF-α and IL-6 in prognostic assessments for ICH patients.

Research focusing on subarachnoid hemorrhage has shown that CSF inflammatory cytokines could be valuable prognostic biomarkers [43–45]. Serum levels of inflammatory cytokines like IL-4, IL-6, and IL-10 in ICH patients are significantly correlated with functional outcomes, and modulating these cytokines may improve prognosis [13, 25, 26, 46]. In our study, we investigated the link between CSF inflammatory cytokine levels and ICH prognosis. Six months after hospital discharge, we found that admission levels of IL-6, IL-8, IL-10, and TNF-α were significantly higher in patients with poor prognoses compared to those with favorable outcomes. This suggests that these cytokines not only affect ICH prognosis but also that elevated admission levels of IL-6, IL-8, IL-10, and TNF-α in the CSF indicate a higher risk of adverse outcomes. This supports the idea that reducing the neuroinflammatory response and cytokine expression post-ICH could lessen cerebral tissue damage [47–50] and improve patient prognosis. Furthermore, our analysis using ROC curves suggests that CSF levels of IL-6, IL-8, IL-10, and TNF-α could be effective prognostic indicators for ICH.

This study acknowledges several limitations. First, the ICH cohort primarily consisted of elderly individuals, while the control group was largely younger. This age disparity could potentially introduce a bias related to age-related factors, considering the limited availability of suitable cases. Additionally, due to variations in the timing of CSF collection for ICH patients, our analysis was restricted to the initial CSF samples collected upon hospital admission. This constraint limits our findings’ applicability in comprehensively assessing disease severity and prognosis over time. Another limitation is the absence of analyses of inflammatory cytokines in concurrent blood samples. This, along with the study’s relatively small sample size and the need for validation of our findings through an independent cohort, highlights areas that require further investigation. In light of these considerations, future research plans include expanding the number of clinical cases and conducting a more extensive, multicenter study for a more thorough validation of the results.

## Conclusions

Our study reveals that the CSF levels of IL-6, IL-8, IL-10, and TNF-α in ICH patients are inversely related to their GCS scores. Additionally, the admission levels of these cytokines in the CSF were significantly higher in patients with a poorer prognosis at six months post-discharge compared to those with favorable outcomes. This suggests that CSF inflammatory cytokine levels could be crucial in assessing the severity of cerebral injury and predicting outcomes in ICH patients. These findings provide valuable insights for clinical diagnosis and treatment strategies in ICH.

## Data Availability

All data reported in this manuscript will be made available from the corresponding author (Qinghai Shi (email: shiqinghai@aliyun.com)) on reasonable request.
